# Evaluation of different glycerol fed-batch strategies in a lab-scale bioreactor for the improved production of a novel engineered β-fructofuranosidase enzyme in *Pichia pastoris*

**DOI:** 10.1007/s11274-024-04027-6

**Published:** 2024-05-31

**Authors:** Gerhardt Coetzee, María del Prado García-Aparicio, Catharine Elizabeth Bosman, Eugéne van Rensburg, Johann Ferdinand Görgens

**Affiliations:** 1 Department of Chemical Engineering, Stellenbosch University, Private Bag X1, Matieland, Stellenbosch, 7602 South Africa; 2 The Centre for Energy, Environmental and Technological Research, Department of Energy, Avda Complutense 40, Madrid, 28040 Spain

**Keywords:** Fed-batch, DO-stat, Constant feed, Glyceraldehyde-3-phosphate dehydrogenase promoter, *Pichia pastoris*, β-fructofuranosidase enzyme

## Abstract

The β-fructofuranosidase enzyme from *Aspergillus niger* has been extensively used to commercially produce fructooligosaccharides from sucrose. In this study, the native and an engineered version of the β-fructofuranosidase enzyme were expressed in *Pichia pastoris* under control of the glyceraldehyde-3-phosphate dehydrogenase promoter, and production was evaluated in bioreactors using either dissolved oxygen (DO-stat) or constant feed fed-batch feeding strategies. The DO-stat cultivations produced lower biomass concentrations but this resulted in higher volumetric activity for both strains. The native enzyme produced the highest volumetric enzyme activity for both feeding strategies (20.8% and 13.5% higher than that achieved by the engineered enzyme, for DO-stat and constant feed, respectively). However, the constant feed cultivations produced higher biomass concentrations and higher volumetric productivity for both the native as well as engineered enzymes due to shorter process time requirements (59 h for constant feed and 155 h for DO-stat feed). Despite the DO-stat feeding strategy achieving a higher maximum enzyme activity, the constant feed strategy would be preferred for production of the β-fructofuranosidase enzyme using glycerol due to the many industrial advantages related to its enhanced volumetric enzyme productivity.

## Introduction

Fructooligosaccharides (FOS) are fructans naturally occuring in certain plants, which include asparagus, sugar beet, onion and Jerusalem artichoke, and it can also be synthesised via various microorganisms, which include *Bacillus macerans* and *Arthrobacter* sp. (bacteria) as well as *Aspergillus niger*, *A. japonicus* and *Aureobasidium pullulans* (fungi) (Singh et al. 2017; Khangwal and Shukla 2019). FOS are considered prebiotic additives and are associated with various health benefits such as reduced cholesterol, enhanced mineral absorption in the gut and bifidogenic effects, and it can furthermore also be utilised as low calorie sweeteners and organoleptic enhancers in food products (Nobre et al. 2022; Costa et al. 2022; Dou et al. 2022). Thus, as a result of the various benefits, prebiotics have garnered increasing attention which is evident by the increase in the market size – in 2015, the global prebiotics market was estimated at about $2.90 billion and due to commercialization efforts it is expected to more than triple by the year 2025, reaching an estimated $10.55 billion (Mano et al. 2018).

FOS molecules are produced by the transfructosylation of sucrose (GF), facilitated by the enzyme β-fructofuranosidases (FFase; EC 3.2.1.26) (Yun 1996). Industrial biotransformation of sucrose to FOS is catalysed by the β-fructofuranosidase enzyme (fopA) from *Aspergillus niger* ATCC 20,611, using a 50–60% (w/v) sucrose substrate at temperatures of between 50 °C and 60 °C (Li et al. 2014; Trollope et al. 2015; Coetzee et al. 2020). FOS consist mainly of 1-kestose (GF_2_), nystose (GF_3_) and 1^F^-fructofuranosyl-nystose (GF_4_) (Yun 1996). Glucose and GF_2_ are the initial products resulting from the sucrose-to-FOS biotransformation and as the reaction progresses, the levels of GF_3_ and GF_4_ increase with a concomitant decrease in GF_2_ (Trollope et al. 2015). Glucose accumulation has previously been shown to inhibit enzyme activity (Nishizawa et al. 2001), limiting the maximum FOS yield to approximately 55–60% (Henderson et al. 2012; Pérez-Cruz et al. 2017; Coetzee et al. 2020). In addition, the stability of the enzyme over the long term is also comprised at temperatures exceeding 50 °C, despite immobilization efforts (Bedzo et al. 2019). Conventional approaches to improve FOS production have mostly been focused around screening for enzymes with enhanced transfructosylating activity (Ghazi et al. 2007; Coetzee et al. 2022) and process development for both enzyme as well as FOS production (Liu et al. 2013; Pérez-Cruz et al. 2017; Coetzee et al. 2020). In more recent years, molecular techniques for altering or improving enzyme function have also received attention (Trollope et al. 2015; Zhang et al. 2017; Coetzee et al. 2022). For example, a fopA enzyme from *A. niger* has been engineered for increased thermostability, specific activity and reduced glucose inhibition, expressed in *Saccharomyces cerevisiae* (Trollope et al. 2015). Furthermore, Zhang et al. also homologously expressed an engineered fopA enzyme in *A. niger* ATCC 20611 (Zhang et al. 2017). However, despite notable advances being made in terms of enhanced FOS production, literature and data at larger scale are still lacking. Thus, in view of commercialisation of such processes, research pertaining to alternative expression hosts, fed-batch enzyme production, FOS production and scale-up development is still much needed.

The methylotrophic yeast *Pichia pastoris* is recognised as a well-rounded expression system and has successfully been employed as a recombinant host for the expression of a large and increasing number and variety of heterologous proteins, such as *Escherichia coli* phytase, mouse endostatin, HAS-GCSF^m^ fusion protein and human antigen-binding fragment (Chen et al. 2004; Garcia-Ortega et al. 2013; Trollope et al. 2015; Jia et al. 2022). This yeast offers many advantages as heterologous protein expression host, which include eukaryotic post-translational modification, growing to high cell-densities on minimal media (maximisation of product yield), high levels of protein expression and secretion as well as ease of genetic manipulation (Karbalaei et al. 2020; Ergün et al. 2021; Coetzee et al. 2022; Pan et al. 2022).

The two main promotors used in *P. pastoris* for recombinant protein expression are alcohol oxidase (*AOX1*) and glyceraldehyde-3-phosphate dehydrogenase (*GAP*). The *GAP* promoter offers a robust constitutive expression of heterologous proteins while avoiding the use of methanol necessary for induction when using the *AOX1* promotor (Çalık et al. 2015). Fed-batch fermentations with the *GAP* promoter usually consist of two phases, namely an initial glycerol batch phase (GB) followed by a glycerol fed-batch phase (GFB) where the glycerol substrate is fed continuously to the culture at a predetermined rate. The feeding methods used during the fed-batch phase could either be DO-stat feed, constant feed or exponential feed (Çalık et al. 2015). The DO-stat regime has the main advantage of maintaining the dissolved oxygen (DO) tension at a level that would sustain an active, healthy culture for extended periods of time. That is, avoiding oxygen-limited growth where oxygen demand exceeds the system’s ability to supply, thus leading to hypoxic conditions that affect cell viability and product formation. The traditional DO-stat feeding strategy has widely been considered as one of the most simple and effective methods to achieve high cell-densities, and to mitigate dynamic changes during substrate feeding (Gao and Shi 2013; Jia et al. 2021). Exponential feed has the advantage of maintaining a constant specific growth rate (µ), which has been directly or indirectly linked to protein production (Potvin et al. 2012; Çalık et al. 2015). Constant feed fermentations have also been used to successfully express various proteins (Goodrick et al. 2001; Zhang et al. 2007; Baumann et al. 2008). However, there is considerable variation in the constant feed rates being employed in recombinant protein production via *P. Pastoris*, i.e., feed rates from 4 to 161 g/L depending on the protein produced; thus, the effect of constant feed on productivity of a bioprocess merits further investigation (Çalık et al. 2015). Furthermore, both exponential and constant feed are examples of feed forward control, which means both feeding strategies are based on the cell growth models of the specific organism (Jia et al. 2022). The specific growth rate for these feeding strategies are not controlled and the growth characteristics of the cultures may change throughout the cultivation process which requires the culture’s feed to be restricted below its optimum specific growth rate (Dietzsch et al. 2011).

In this study, the production levels of two β-fructofuranosidase enzymes used in the synthesis of fructooligosaccharides (FOS) will be investigated. A native (GAPfopA) as well as a novel engineered (GAPfopA_V1) enzyme (Trollope et al. 2015) will be investigated and compared in larger bench-scale bioreactors, using different fed-batch feeding strategies – the DO-stat and the constant feed strategies with glycerol as carbon source. A *P. pastoris* expression system under control of the *GAP* promotor will be employed in the current study due to the industrial advantages of using glycerol over methanol (safety and acceptability for food-production).

## Materials and methods

### Microbial strains, media and plasmids

*Escherichia coli* DH5α [fhuA2Δ(argF-lacZ)U169 phoAglnV44 Φ80Δ (lacZ)M15 gyrA96 recA1 relA1 endA1 thi-1hsdR17] (New England Biolabs, Midrand, South Africa) served as host for plasmid amplification. Cells were grown at 37 °C in low salt Luria Bertani broth supplemented with 25 µg/mL zeocin. The *Pichia pastoris* strain (GAPfopA) containing the native codon-optimised gene (ATUM, Newark, USA) under control of the glyceraldehyde-3-phosphate dehydrogenase (*GAP*) promoter was obtained from the culture collection of the Department of Chemical Engineering, Stellenbosch University, South Africa (Trollope et al. 2015). The *P. pastoris* strain DSMZ 70382 (CBS704) was selected as expression host for the production of the engineered FFase enzyme (Table 1). *P. pastoris* cells were grown in yeast peptone dextrose (YPD) medium consisting of 1% yeast extract, 2% peptone and 2% glucose, supplemented with sorbitol and zeocin, as appropriate (100 to 1000 µg/mL). Solid media were further supplemented with agar (15 g/L). For enzyme expression in shake flasks, *P. pastoris* transformants were cultivated in buffered glycerol complex medium (BMGY) consisting of 1% glycerol, 1% yeast extract, 2% peptone, 1.34% yeast nitrogen base, 4 × 10^− 5^ % biotin and 100 mM ptassium phosphate buffer (pH 6). Zeocin was purchased from Melford Laboratories Ltd (Chelsworth, UK). All other chemicals were purchased from Sigma-Aldrich® (South Africa) or Merck (South Africa) with the yeast nitrogen base obtained from Becton, Dickinson and Company (Franklin Lakes, NJ, USA).

**Table 1:** Microbial strains in this study

Strains and plasmids	Genotype or construct	Source
*Pichia pastoris* DSMZ 70382	Type strain	Leibniz Institute DSMZ – German Collection of microorganisms and cell cultures
pJ905-*fopA*	*Sh ble GAP*_*P*_ *-xln2*_*S*_ *-fopA-AOX1*_*T*_	Department of Chemical Engineering, Stellenbosch University, South Africa
pJ905-*fopA_V1*	*Sh ble GAP*_*P*_ *-xln2*_*S*_ *-fopA_V1-AOX1*_*T*_	This study; ATUM (Menlo Park, USA)
GAPfopA	*P. pastoris* DSMZ 70,382 with pJ905-*fopA*	Department of Chemical Engineering, Stellenbosch University, South Africa
G100, G250, G500, G1000	*P. pastoris* DSMZ 70,382 with pJ905-*fopA_V1*	This study

### DNA cloning and yeast transformation

The four amino acid substitution (F140Y-A178P-G321N-Q490S) variant of the *Aspergillus japonicus* fopA FFase (GenBank accession number AB046383) has comprehensively been described in a previous work (Trollope et al. 2015). The fopA_V1 coding sequence, fused to the *Trichoderma reesei* endoxylanase 2 (*xln2*) secretion signal, was cloned from the pJ227 cloning vector as a 2027 bp EcoRI-XhoI fragment into the *P. pastoris* expression vector pJ905, harbouring the *GAP* promoter (ATUM, Newark, USA). The pJ905_fopA_V1 plasmid was linearised with XmaJI (Thermo Fisher Scientific, Waltham, USA) prior to yeast transformation (Lin-Cereghino et al. 2005). The electroporation was carried out in electroporation cuvettes (gap, 2.0 mm) in a Gene Pulser® II electroporator (Bio-Rad Laboratories, Hercules, CA, USA) with charging voltage of 1500 V, resistance of 200 Ω and capacitance of 25 µF. After the electroporation, the cells were incubated in YPD medium supplemented with 1 M sorbitol for 3 h. Thereafter, cells were plated on selective media at 30 °C for up to 3 days until colonies had formed.

Transformants were transferred to YPD plates consisting of 1% yeast extract, 2% peptone, 2% glucose and 1.3% agar following which they were re-streaked on YPD plates with increasing concentrations of zeocin. Positive transformants were further confirmed by colony PCR with fopA_V1-specific primers. The sequences of the primers were 5’-ACGCATGTCATGAGATTATTGG-3’and 5’-GCAAATGGCATTCTGACATCC-3’ (Inqaba Biotechnical Industries (Pty) Ltd, Pretoria, South Africa).

### Transformant screening

Transformant screening was conducted according to the method outlined by Krettler et al. (Krettler et al. 2013). Single colonies of the transformants were inoculated into test tubes containing 5 mL of BMGY medium and grown overnight on a rotating wheel, while maintaining the temperature at 30 °C. Following this, the respective cultures were inoculated into 500 mL baffled shake flasks, each containing 100 mL of BMGY medium. The cultures were incubated overnight at 30 °C, with an agitation speed of 200 rpm. After the cultures had reached an optical density greater than or equal to 10 (OD_600_ > = 10), the fractional volume of the culture to be harvested was calculated for inoculation of the small-scale expression culture (100 mL of BMGY in 500 mL baffled flasks) at OD_600_ = 1. This volume was centrifuged (3000 g for 3 min) and resuspended in YP medium consisting of 1% yeast extract and 2% peptone, after which it was inoculated into the baffled flasks and incubated on an orbital shaker (200 rpm) for 72 h at 30 °C. Samples were collected in 24 h time-intervals and the glycerol concentration was maintained at 1% glycerol, by adding a volume of glycerol equal to what was removed during each sample. The supernatant was maintained at 4 °C for determination of enzyme activity. These experiments were conducted in triplicate to allow for the determination of standard deviation.

### Bioreactor cultivations

*P. pastoris* cultivations were carried out in BioFlo® 110 bioreactors (New Brunswick Scientific Co. Inc., Edison NJ, USA) fitted with a 10 L glass reactor vessel (8 L working volume). Temperature was controlled using a heating jacket and cooling coil and the reactor was fitted with a combination glass pH electrode and polarographic dissolved oxygen (DO) probe (all Mettler Toledo, Sandton, South Africa). The BioCommand® (V 3.30 Plus) software (New Brunswick Scientific Co. Inc.) was used to monitor DO, temperature, pH, agitation and feed rate control. Fermentations were conducted according to the method outlined in the *Pichia* Fermentation Guidelines (Invitrogen Corporation 2002) (Thermo Fisher Scientific, Waltham, MA, USA) with modifications as noted below. For the bioreactor cultivations, starter cultures were prepared by inoculating several colonies from YPD agar plates into test tubes containing 8 mL of buffered minimal glycerol (BMG) culture medium consisting of 1.34% yeast nitrogen base, 1% glycerol, 1.64 µM biotin and 100 mM potassium phosphate buffer (pH 6.0). The cultures were incubated for 24 h at 30 °C. Following this incubation period, 2 L Erlenmeyer shake flasks containing 400 mL of fresh BMG medium were inoculated to a final biomass concentration equivalent to OD_600_ = 0.1, measured using a spectrophotometer (Biochrom WPA Lightwave II, Harvard Bioscience, Holliston, MA, USA). These cultures were grown for a further 18 h on an orbital shaker (200 rpm) at 30 °C, until a biomass concentration equal to an OD_600_ of between 6 and 8 was reached. The entire volume of these cultures was used to inoculate the bioreactor to a final volume of 4 L, resulting in an initial biomass concentration equal to an OD_600_ of between 0.6 and 0.8 at the start of the batch phase.

Basalt salt medium (BSM), supplemented with 1% casein hydrolysate and PTM_1_ trace salts consisting of (per 1 L) 6.0 g CuSO_4_∙5H_2_O, 0.08 g NaI, 3.0 g MnSO_4_∙H_2_O, 0.2 g Na_2_MoO_4_∙2H_2_O, 0.02 g H_3_BO_3_, 0.5 g CoCl_2_, 20.0 g ZnCl_2_, 65 g FeSO_4_∙7H_2_O, 0.2 g biotin and 5.0 ml H_2_SO_4_ was used as culture medium in the bioreactors. Cultivations were carried out at 30 °C and the culture pH was maintained at pH 5 through the addition of 28% ammonium hydroxide (Sigma-Aldrich®). Atmospheric air was sparged at an aeration rate of 1.0 vvm to maintain the dissolved oxygen (DO) tension above 30% of saturation (Çalık et al. 2015). Due to the possibility of the rate of oxygen consumption exceeding the rate of oxygen transfer at high biomass concentrations, the agitation rate (200 to 1000 rpm) was cascaded to the air flow rate, followed by the sparging of pure O_2_, in order to maintain aforementioned DO tension.

Two glycerol fed-batch strategies were used in this study, namely a DO-stat and a constant feed strategy. During the DO-stat strategy, a solution of 50% (w/v) glycerol (Scienceworld, South Africa) was supplied to the culture at a rate of 18.15 mL/h/L of initial fermentation volume. Since depletion of the glycerol carbon source would result in an increase in the DO, the pumps automatically fed the culture at the abovementioned rate when the DO exceeded 35% of saturation. Conversely, since the DO would decrease with a supply of carbon to the culture, the same threshold of 35% of saturation was used to switch the pumps off to prevent overfeeding of the culture. According to existing literature on recombinant protein production in *P. pastoris*, constant feeding rate is a parameter that has yet to be optimized – literature values for constant carbon feeding rate vary anything from 4 to 161 g/L. In this study, for the constant feeding strategy a solution of 50% (w/v) glycerol was supplied to the culture at a rate of 18.15 mL/h/L of initial fermentation volume, which commenced at the end of the batch phase. In each strategy, the cultivations were terminated when a final volume of 8 L was reached. Samples were collected throughout the cultivation process, after which they were centrifuged (13,000 rpm for 3 min), filtered (0.22 μm syringe filters), and analyzed for dry cell weight (DCW) concentration, volumetric enzyme activity and glycerol concentration.

### Analytical methods

#### Dry cell weight (DCW) concentration

DCW concentration was determined by first centrifuging (Prism™ Microcentrifuge, Labnet International, Edison, NJ, USA) 2 mL of culture supernatant (13,000 rpm for 3 min). The sample was then resuspended in distilled water (dH_2_O), centrifuged again and dried in an oven at 60 °C for approximately 24 h (until weight stabilized) following which it was converted to DCW per liter of whole broth.

#### Enzyme activity assay

The activity of the FFase enzyme was determined using 100 g/L sucrose (Merck) as substrate prepared in 50 mM citrate phosphate buffer (pH 5.5). The substrate solution (0.75 mL) was equilibrated at 40 °C for 10 min prior to adding the enzyme-containing culture supernatant (0.25 mL) to give a final concentration of 25% (v/v). After incubation at 40 °C for 60 min, the reaction was terminated by addition of 35% (w/v) perchloric acid (PCA) to a final concentration of 2.14% (v/v) followed by the addition of 7 M KOH to precipitate the proteins prior to high-performance liquid chromatography (HPLC) analysis (Hidaka et al. 1988). Negative controls consisted of all the assay constituents except either sucrose or the enzyme which was replaced with 50 mM citrate phosphate buffer (pH 5.5).

The concentration of glucose liberated during the assays was indicative of enzyme activity. A unit of FFase enzyme was defined as the amount of enzyme required to produce 1 µmol glucose per minute, under the described conditions (Hidaka et al. 1988). The glucose concentration was determined using a Dionex UltiMate 3000 system (Thermo Fisher Scientific, Waltham, MA, USA) equipped with a Coulochem III electrochemical detector controlled by Chromeleon™ 6.8 Chromatography Data System software (Thermo Fisher Scientific). The HPLC was fitted with a CarboPac PA1 (4 × 250 mm) analytical column coupled to a PA1 (4 × 50 mm) guard column (Thermo Fisher Scientific). Samples were injected at a volume of 10 µL and eluted according to the method described by van Wyk et al. (van Wyk et al. 2013).

#### Glycerol concentration

The glycerol concentration was analysed by HPLC (Finnigan Surveyor, Thermo Fisher Scientific) using a Rezex RHM-Monosaccharide column (Phenomenex, Torrance, CA, USA) fitted with a guard column. A Refractive Index (RI) detector (Thermo Fisher Scientific, Finnigan Surveyor) was used to quantify the glycerol by integrating the area under the peak of the eluted substance, confirmed using a series of standards of a known range of concentrations. The Rezex RHM-Monosaccharide column was maintained at 60 °C. A 5 mM H_2_SO_4_ solution was used as mobile phase at a flow rate of 0.6 mL/min for 25 min. Samples were acidified with 10% H_2_SO_4_ to a final concentration of 0.5% (v/v) and filtered through 0.22 μm nitrocellulose filters (Membrane Solutions, Kent, WA, USA) prior to analysis.

#### SDS-PAGE analysis

To determine enzyme production, SDS-PAGE was performed according to the method described by Laemmli (Laemmli 1970). As per this method, a separating gel with an 8% polyacrylamide concentration and a stacking gel with 5% concentration was used. The gels were silver stained according to the method outlined by Merril et al. (Merril et al. 1981).

### Calculations and statistical analysis

Maximum growth rate (µ_max_) was determined graphically by plotting the natural logarithm of the biomass concentration (g/L) during batch phase vs. fermentation time. The maximum growth rate was then determined from the gradient of the exponential growth phase. The biomass yield (*Y*_XS_) and product yield (*Y*_PS_) were determined by plotting the total biomass (g) and total enzyme activity (U), respectively, against the mass of substrate consumed (g), and the slope of these plots were subsequently calculated. Biomass productivity (*Q*_X_) was determined using Eq. 1, where *X* denotes the total biomass (g) at a specified time, *t* (h) and volume, *V* (L).

1 $${Q}_{x}=\frac{X}{V\cdot t}$$

Volumetric productivity (*Q*_P_) was determined according to Eq. 2, where *P* denotes the total product (U) at any time, *t* (h) and volume, *V* (L).

2 $$Q{}_{P}=\frac{P}{V\cdot t}$$

For all experimental data, the means of either independent triplicates (transformant screening) or two replicates (bioreactor runs) were determined. Standard deviations were calculated and provided to indicate variability in the data.

## Results

### Screening of *P. pastoris* transformants

**Fig. 1:**
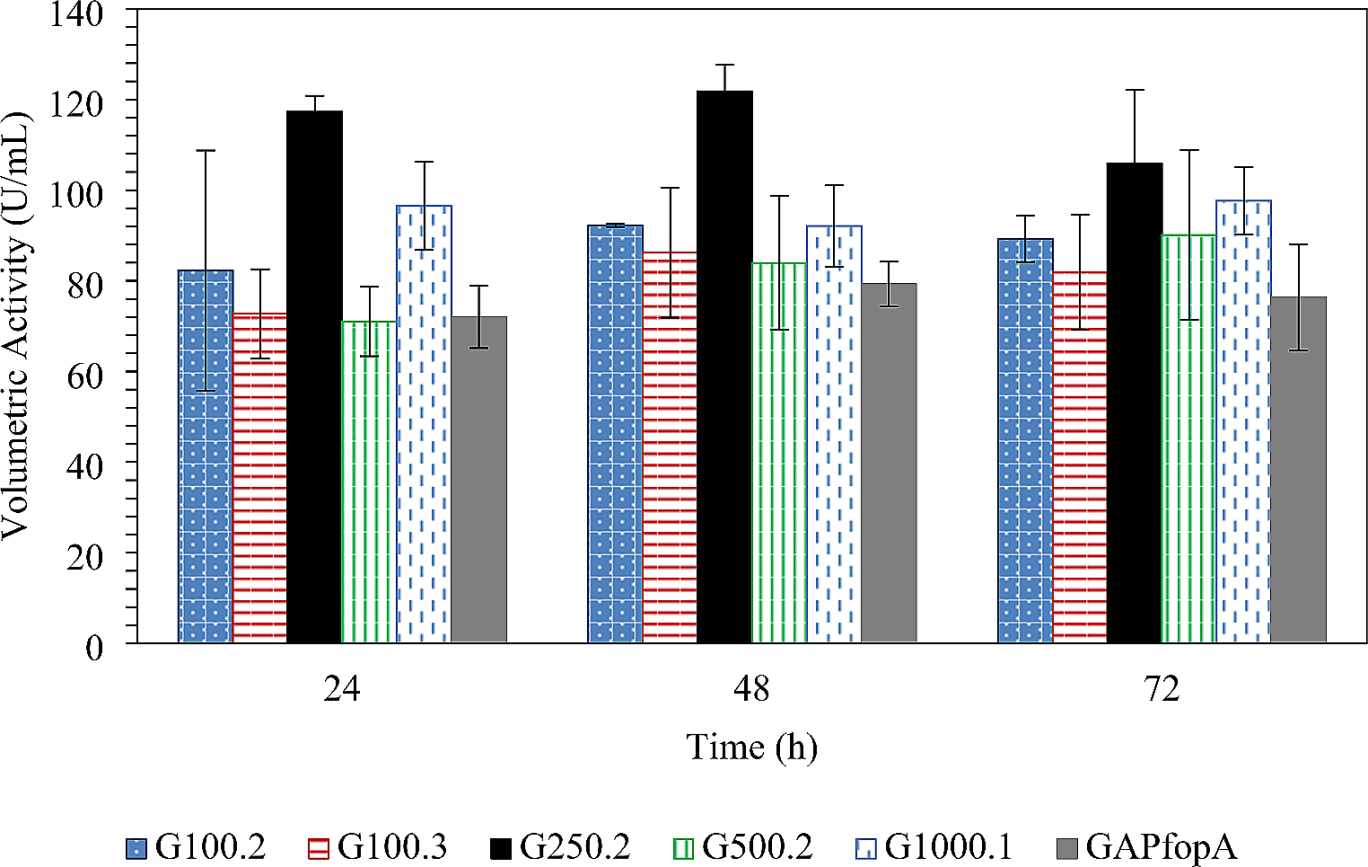
Volumetric enzyme activity of *P. pastoris* transformants containing the *GAPfopA_V1* gene relative to the control with the *GAPfopA* gene in shake flasks. Cultivations were performed at 30 °C over 72 h at an agitation rate of 200 rpm (Error bars denote the standard error of triplicate experiments)

Selective YPD plates containing zeocin were used to screen for *P. pastoris* strains transformed with the *fopA_V1* gene. Thereafter, positive transformants were confirmed using PCR. Seven transformants were confirmed and subsequently screened in shake flasks to identify the strain producing the highest volumetric activity compared to the GAPfopA reference strain (Fig. 1). Despite confirmed transformation of the *fopA_V1* gene using PCR, five of the seven transformants exhibited marked enzyme activity, however, no activity was detected for strains G100.1 and G250.3. Strain G250.2 exhibited the highest activity of 121.8 ± 6.0 U/mL after 48 h, which was 35% higher than that of the reference strain (GAPfopA) which produced a maximum activity of 79.3 ± 4.93 U/mL within the same timeframe (Fig. 1). As a result, Strain G250.2 was selected for further study and will henceforth be referred to as strain GAPfopA_V1. Refer to Table 1 for more information on the strains considered in this study.

### Bioreactor cultivations

**Fig. 2:**
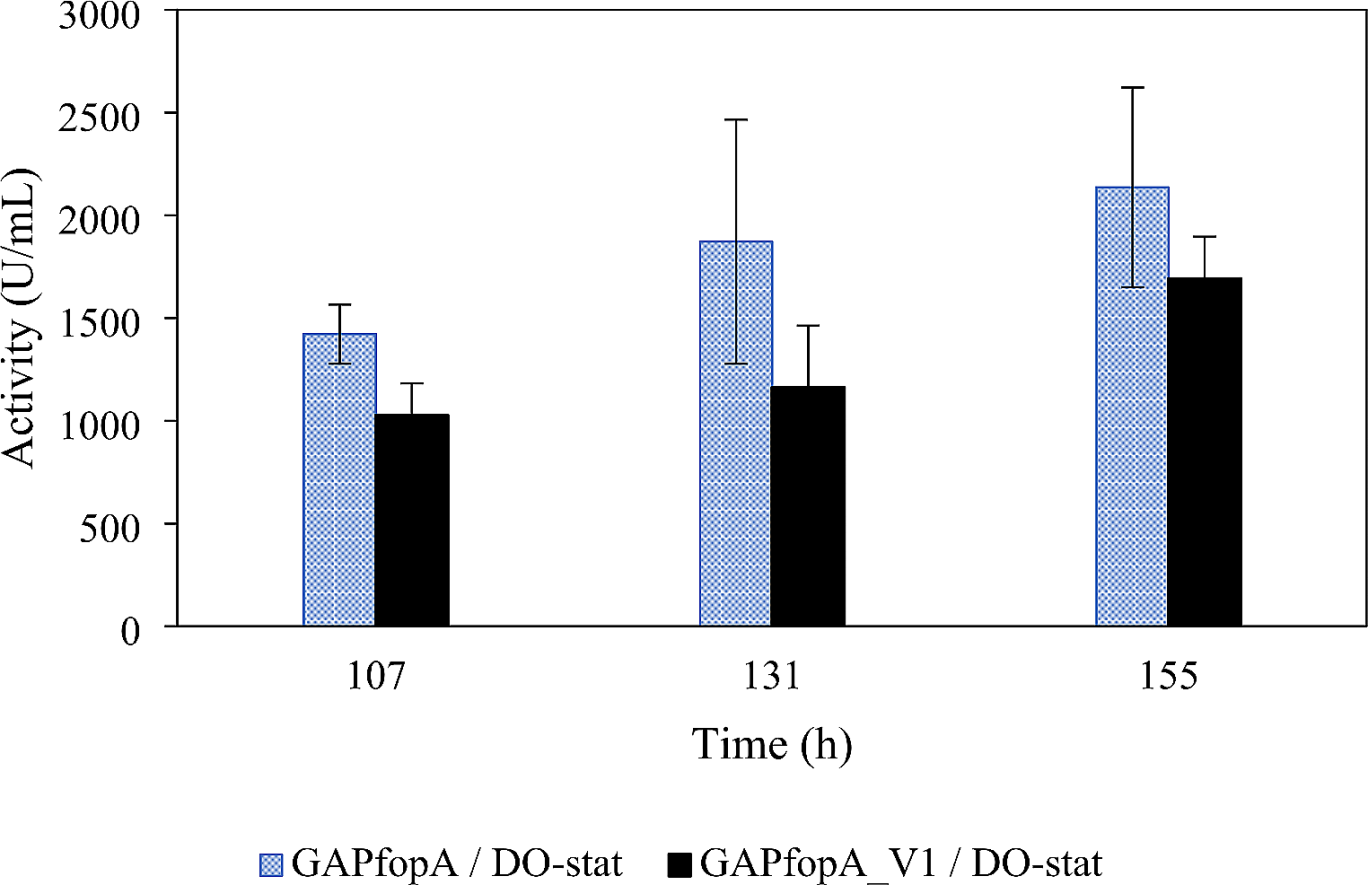
Volumetric enzyme activities over time for DO-stat cultivations for the GAPfopA and GAPfopA_V1 enzymes. Cultivations performed at 30 °C, pH 5 and 30% dissolved oxygen (Error bars denote standard deviations with *n* = 2)

**Fig. 3:**
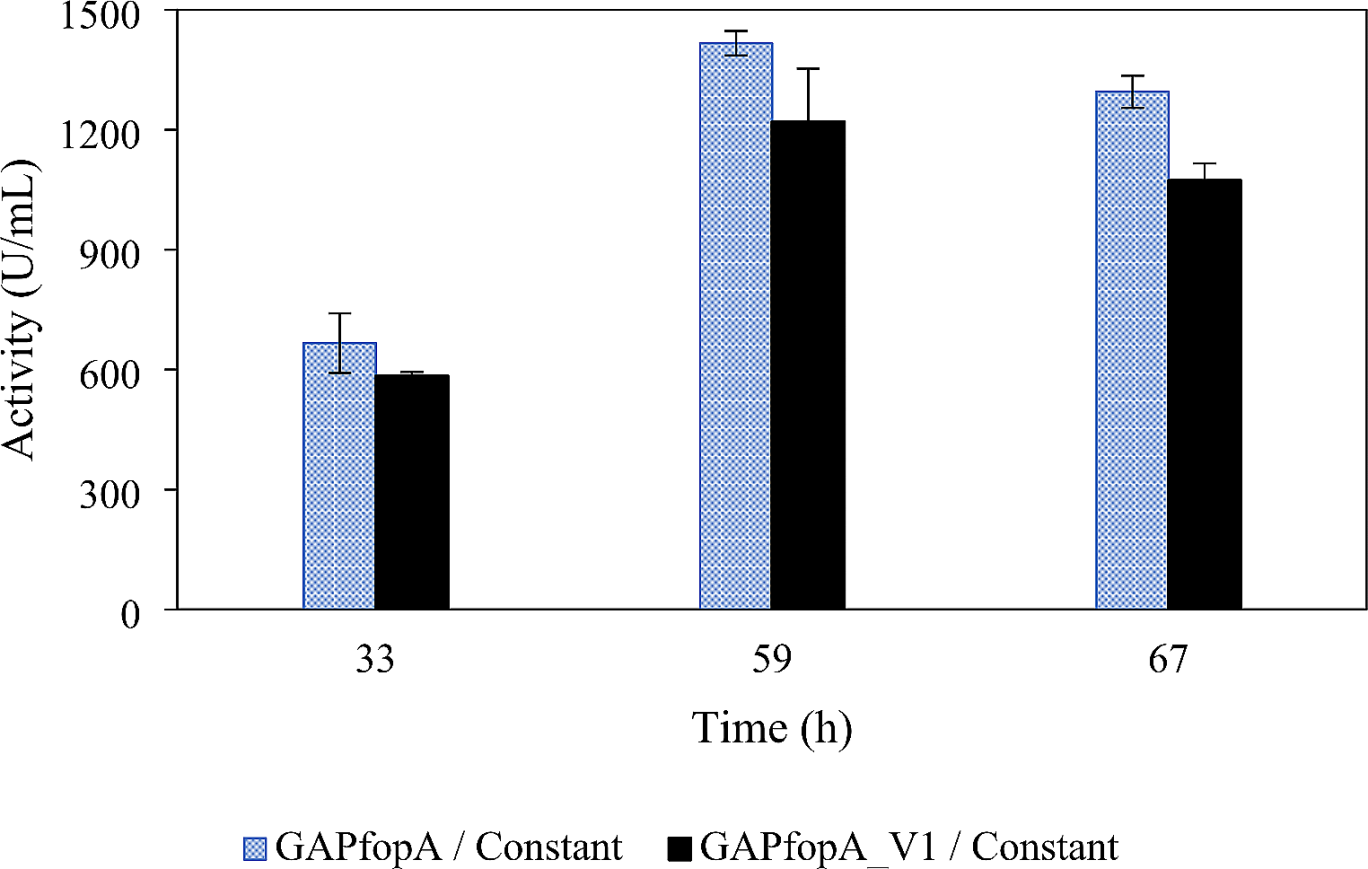
Volumetric enzyme activities over time for constant feed cultivations for the GAPfopA and GAPfopA_V1 enzymes. Cultivations performed at 30 °C, pH 5 and 30% dissolved oxygen (Error bars denote standard deviations with *n* = 2)

**Fig. 4:**
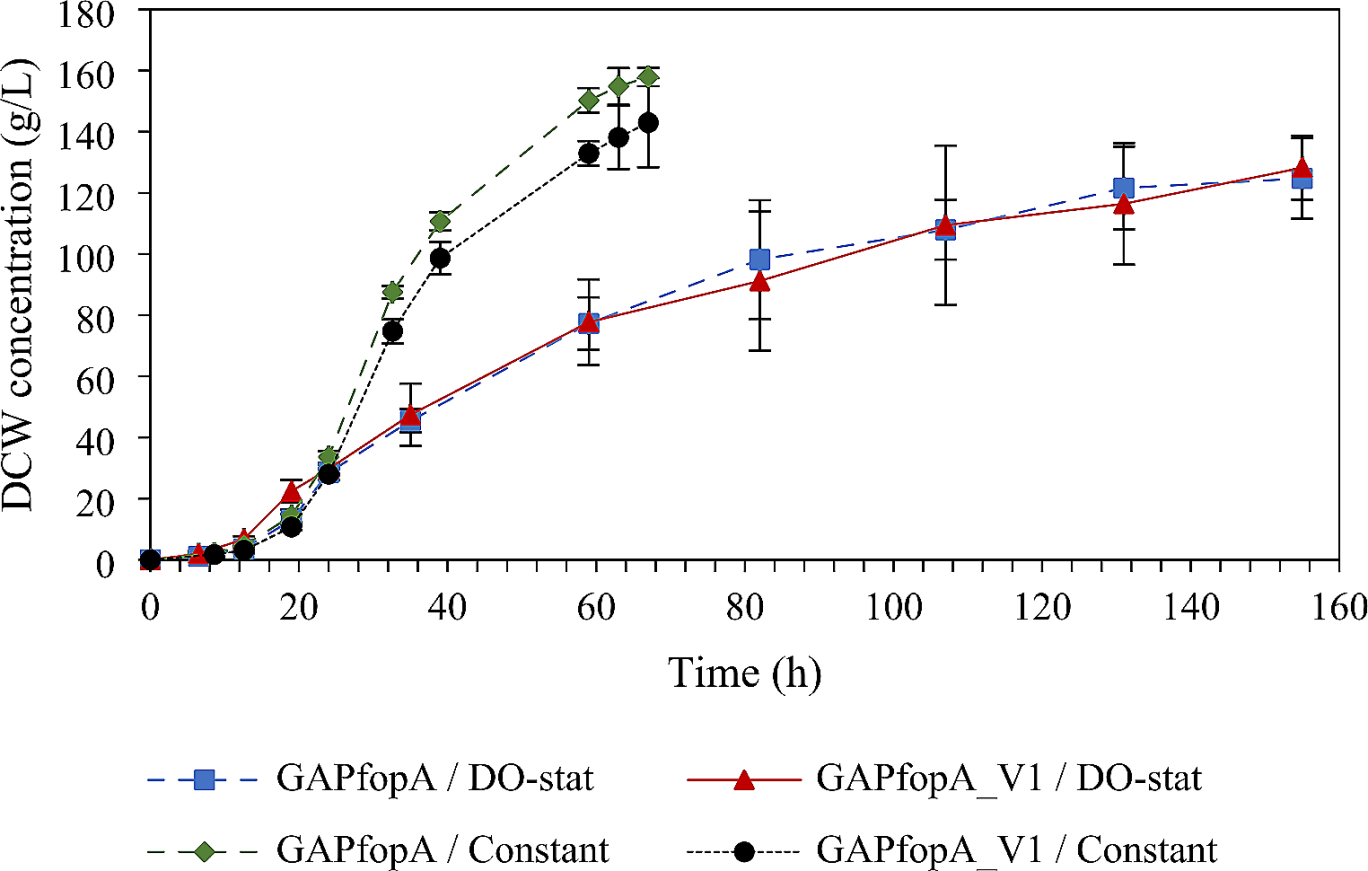
Dry cell weight (DCW) concentrations over time for DO-stat as well as constant feed cultivations for the GAPfopA and GAPfopA_V1 enzymes. Cultivations performed at 30 °C, pH 5 and 30% dissolved oxygen (Error bars denote standard deviations with *n* = 2)

The cultivation and enzyme production results for strains GAPfopA and GAPfopA_V1, grown in bioreactors using either a DO-stat or a constant feed strategy are summarised in Table 2. The maximum volumetric activity achieved during the cultivation period was noted for both feeding strategies. For the DO-stat feeding strategy the maximum activity was recorded at 155 h (Fig. 2), while the maximum activity for the constant feeding strategy was recorded at 59 h (Fig. 3). The maximum specific growth rate (µ_max_) during the batch phase and the biomass concentration were comparable for both strains operating under both feeding strategies (Fig. 4), i.e., DO-stat and constant feed. Therefore, it could be inferred that the respective cultures were in similar metabolic states from the start of the fed-batch phase.

**Table 2:** Fermentation results for the two strains, GAPfopA and GAPfopA_V1, cultivated in 10 L bioreactors fed with two different substrate feeding strategies, DO-stat and constant feed

Feed method	Enzyme	Batch phase		Fed-batch phase					
		µ_max_ (h^− 1^)	DCW conc. (g/L)	Volumetric productivity, *Q*_P_ (×1000)^a^ (U/L/h)	Volumetric activity^a^ (U/mL)	Product yield, *Y*_PS_ (U/g)	DCW conc.^a^ (g/L)	Biomass yield, *Y*_XS_ (g/g)	Biomass productivity, *Q*_X_^a^ (g/L/h)
DO-stat	GAPfopA	0.21 ± 0.02	27.89 ± 0.01	13.74 ± 5.27	2129.25 ± 816.39	11485.15 ± 7697.35	119.73 ± 1.99	0.49 ± 0.00	0.77 ± 0.01
	GAPfopA_V1	0.19 ± 0.04	27.32 ± 1.13	10.88 ± 1.37	1686.91 ± 212.29	9892.10 ± 865.36	118.39 ± 10.69	0.47 ± 0.01	0.76 ± 0.07
Constant	GAPfopA	0.24 ± 0.02	26.69 ± 0.08	23.96 ± 0.44	1413.36 ± 26.18	5670.00 ± 592.41	133.86 ± 3.92	0.55 ± 0.03	2.27 ± 0.07
	GAPfopA_V1	0.20 ± 0.03	27.99 ± 2.42	20.72 ± 2.30	1222.70 ± 135.69	4587.50 ± 74.10	132.44 ± 12.95	0.54 ± 0.03	2.24 ± 0.22

Mean ± standard deviation; *n* = 2

^a^ Analyses were done at the maximum volumetric activity, i.e., at 155 h for DO-stat and 59 h for constant feed, respectively

**Fig. 5:**
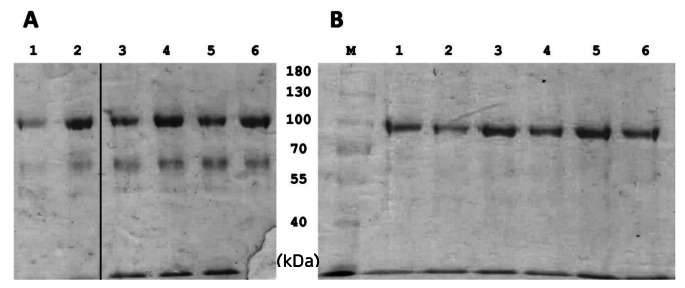
SDS-PAGE of the crude supernatant of the cultivations of two different feeding strategies: (A) constant feed with odd-numbered lanes representing GAPfopA_V1 and even-numbered lanes GAPfopA. Lanes 1 and 2 (33 h), lanes 3 and 4 (59 h) and lanes 5 and 6 (67 h). (B) DO-stat feed with odd-numbered lanes representing GAPfopA and even-numbered lanes GAPfopA_V1. Lanes 1 and 2 (107 h), lanes 3 and 4 (131 h) and lanes 5 and 6 (155 h). Lane M represents the molecular weight marker. Black line in (A) indicates removal of an empty lane on gel

The rate of enzyme production was higher for the constant feeding strategy cultivations, due to substrate being fed at a higher rate. As shown in Table 2, this was reflected in the volumetric productivity (*Q*_P_) for the constant feed cultivations (23.96 ± 0.44 × 10^3^ U/L/h for GAPfopA and 20.72 ± 2.30 × 10^3^ U/L/h for GAPfopA_V1), which notably exceeded that achieved in the DO-stat cultivations (13.74 ± 5.27 × 10^3^ U/L/h for GAPfopA and 10.88 ± 1.37 × 10^3^ U/L/h for GAPfopA_V1) for both strains. However, the faster rate of enzyme production did not necessarily correlate to maximum volumetric activity. The DO-stat cultivations exhibited considerable variation in the volumetric activity, which is evident through the large standard deviations (Table 2). Nonetheless, despite this variation, the trend still indicated potentially higher volumetric activity for the GAPfopA strain (2129.25 ± 816.39 U/mL) compared to the GAPfopA_V1 strain (1686.91 ± 212.29 U/mL) for the DO-stat feeding strategy (Fig. 2), with the higher volumetric activity also reflected in the higher enzyme production for the GAPfopA strain (Fig. 5b).

Similar to the DO-stat cultivations, the constant feed cultivations also exhibited similar results between the two different strains (Table 2). The batch phase specific growth rate (0.24 ± 0.02 h^− 1^ and 0.20 ± 0.03 h^− 1^), biomass productivity (*Q*_x_; 2.27 ± 0.07 g/L/h and 2.24 ± 0.22 g/L/h), DCW concentration (133.86 ± 3.92 g/L and 132.44 ± 12.95 g/L) as well as the biomass yield (*Y*_xs_; 0.55 ± 0.03 g/g and 0.54 ± 0.03 g/g) were relatively close for GAPfopA and GAPfopA_V1, respectively (Table 2). The constant feed cultivations trend exhibited for volumetric activity of the two strains also reflects the trend exhibited for the DO-stat cultivations with the GAPfopA strain (1413.36 ± 26.18 U/mL) achieving a slightly higher activity than the GAPfopA_V1 strain (1222.70 ± 135.69 U/mL) (Fig. 3). This was also corroborated by higher protein production for GAPfopA (Table 2; Fig. 5a).

When directly comparing the constant feed and DO-stat cultivations, the constant feed cultivations outperformed the DO-stat cultivations on several metrics. The constant feed cultivations achieved more than triple the biomass productivity of the DO-stat cultivations (~ 2.2 g/L/h compared to ~ 0.7 g/L/h), a DCW approximately 14 g/L more than the DO-stat cultivations (Fig. 4) and also a slightly higher biomass yield (maximum of 0.55 ± 0.03 g/g compared to 0.49 ± 0.00 g/g) for both investigated strains (Table 2). However, despite the increased biomass production for the constant feed cultivations, the DO-stat cultivations outperformed the constant feed cultivations in terms of maximum volumetric activity, which was generally higher for the DO-stat cultivations for both the strains (Table 2; Figs. 2 and 3).

**Fig. 6:**
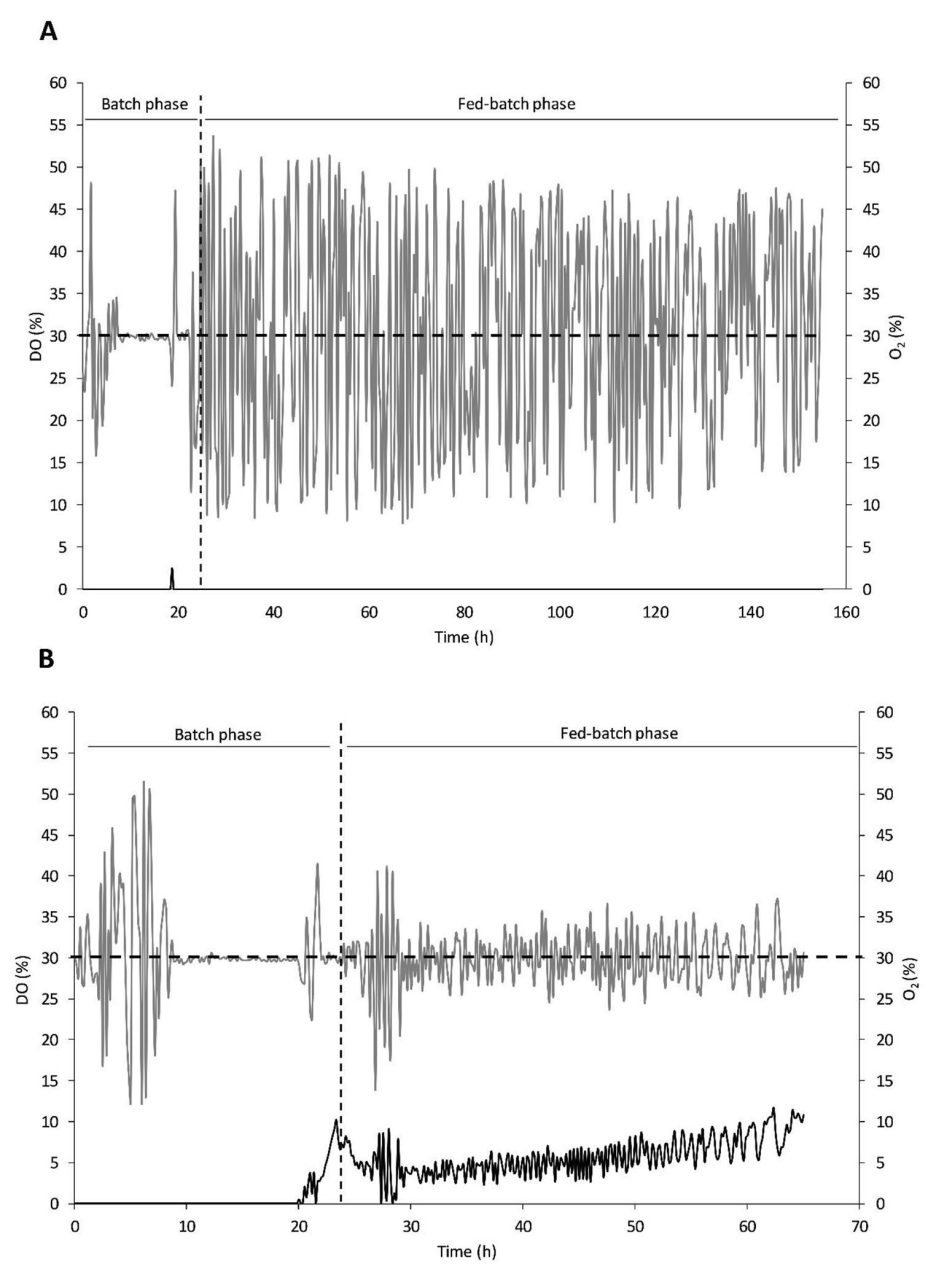
Dissolved oxygen (DO) level (grey) and O_2_ sparged (black) during the (A) DO-stat and (B) constant feed fermentations. The horizotal black dashed line indicates the setpoint value at which the DO is controlled

The fermentation control system was configured to maintain the DO at 30% of saturation irrespective of fed-batch feeding strategy. During DO-stat cultivations, the DO exhibited significant variation from the setpoint during the fed-batch phase, which ranged between approximately 10% and 55% of saturation (Fig. 6a). Conversely, oscillations in the DO were substantially less pronounced during the fed-batch phase where constant feed was applied and was generally maintained between 20% and 40% of saturation (Fig. 6b). However, the greater biomass achieved during constant feed necessitated sparging with pure O_2_ throughout the fed-batch phase to maintain the required DO levels.

## Discussion

As mentioned, the aim of this study was to compare the production of enzyme activity for two different *P. pastoris* strains (GAPfopA and GAPfopA_V1), each harbouring a different version of the fopA enzyme under control of the *GAP* promoter, while employing two different feeding strategies – the DO-stat and constant feed strategies using glycerol as carbon source.

Consistent improvement in relative enzyme production by the two recombinant strains used in this study was not exhibited when transferring from shake flasks to bioreactors. In the shake flasks, however, the GAPfopA_V1 strain (transformant strain G250.2) achieved a noTable 53.6% improvement in volumetric activity compared to the GAPfopA reference strain, increasing activity from 79.3 ± 4.93 U/mL (GAPfopA) to 121.8 ± 6.0 U/mL (GAPfop_V1) (Fig. 1). This result was in contrast to the results obtained for the bioreactor-grown fed-batch cultures where the GAPfopA strain exhibited a higher volumetric enzyme activity and level of protein production (Table 2; Fig. 5a and b) despite the two strains producing similar biomass concentrations in the DO-stat (119.73 ± 1.99 g/L for GAPfopA and 118.39 ± 10.69 g/L for GAPfopA_V1) as well as constant feed (133.86 ± 3.92 g/L for GAPfopA and 132.44 ± 12.95 g/L for GAPfopA_V1) fermentations. In the bioreactors, the GAPfopA_V1 strain had a volumetric enzyme activity 20.8% and 13.5% lower than the native GAPfopA reference strain under DO-stat (Fig. 2) and constant feed (Fig. 3) strategies, respectively. Reproducibility and scalability limitations related to screening in microtitre plates have previously been reported (Hemmerich et al. 2014), suggesting that screening done in microtitre plates are not always reflective of what occurs in a bioreactor. The limitations of microtitre plate screening are mainly ascribed to “edge effects” (Mansoury et al. 2021) and also possibly due to poorly controlled substrate feeding. Screening in microtitre plates and/or shake flasks are typically performed using a pulse feeding regime where an initial excess of substrate is followed by substrate depletion, leading to inconsistent growth conditions. In bioreactors, however, substrate is fed in a more consistent manner with fewer interruptions, which can circumvent substrate depletion. As a result, biomass growth is more consistent, leading to enhanced production of recombinant proteins (Looser et al. 2015). Furthermore, the transformant achieving the highest productivity generally suffers from increased metabolic burden of recombinant protein production; thus, such transformants are not necessarily always the best candidates for bioreactor cultivation (Holmes et al. 2009; Mansoury et al. 2021).

The growth performance of the respective strains was within expected levels, indicating no anomalies in the physiological growth of the strains. The maximum growth rates attained by the strains evaluated during the batch phase were between 0.19 ± 0.04 h^− 1^ and 0.24 ± 0.02 h^− 1^ (Table 2), similar to what has previously been reported for *P. pastoris* using a glycerol carbon source (0.18 h^− 1^ to 0.29 h^− 1^) (Ren et al. 2003; Cos et al. 2005; Looser et al. 2015). The yields achieved in this study ranged between 0.47 ± 0.01 g/g and 0.55 ± 0.03 g/g, also in-line with previously reported literature on *P. pastoris* expression systems (0.43 g/g to 0.62 g/g) (Ren et al. 2003; Cos et al. 2005; Looser et al. 2015). Furthermore, the obtained biomass concentrations of between 118.39 ± 10.69 g/L and 133.86 ± 3.92 g/L (Table 2), were also corroborated by literature-reported data (up to 150 g/L on methanol substrate (Curvers et al. 2001); 200 g/L on glucose substrate (Heyland et al. 2010).

In terms of culture growth performance, constant feed fed-batch outperformed DO-stat fed-batch cultivations (Fig. 3). The constant feed fermentation exhibited higher biomass productivity (maximum of 2.27 ± 0.07 g/L/h for constant feed and 0.77 ± 0.01 g/L/h for DO-stat), biomass yield (maximum of 0.55 ± 0.03 g/g for constant feed and 0.49 ± 0.00 g/g for DO-stat) as well as DCW concentration (maximum of 133.86 ± 3.92 g/L for constant feed and 119.73 ± 1.99 g/L for DO-stat) compared to the DO-stat fermentation. This was mainly attributed to poor feed control in the DO-stat cultivations, stemming from poor DO control. In this study, the DO was controlled via a proportional-integral-derivative (PID) controller, which has previously been found to be limiting in controlling DO in high-cell-density fermentations – this is mainly due to the rapid variations in DO during the latter stages of the fermentation process under DO-stat control (Chung 2000; Ferreira et al. 2012). A study by Chung showed that during the fed-batch phase, feeding either methanol or glycerol, the rate of oxygen transfer and oxygen utilization becomes critically close (Chung 2000). This leads to increased oscillations in the metabolic behaviour of *P. pastoris* and results in controller destabilization and loss of culture productivity (Chung 2000). In DO-stat control, the amount of glycerol fed to the system is based solely on the continuous oscillations in the online DO measurements which leads to lower glycerol concentrations in the system (Jia et al. 2021). Due to the resulting glycerol limitation, cell growth rate has generally been found to be lower under DO-stat control (Gao and Shi 2013; Jia et al. 2021). In the current study the DO level oscillated between ~ 10% and ~ 55% during the DO-stat cultivations (Fig. 6a). This created an artificially wide DO band resulting in intermittent glycerol feeding and, therefore, lower biomass growth compared to that of the constant feed fermentation. A study by Lee et al. evaluated cell-growth during the glycerol fed-batch phase for derepression of the *AOX1* promoter, by feeding glycerol under the DO-stat method (Lee et al. 2003). The feed was controlled within a band consisting of two DO setpoints – a lower setpoint of 10% DO and an upper setpoint of either 30% DO or 50% DO (Lee et al. 2003). As such, feeding was initialized when the DO exceeded 10% and terminated when it reached the upper DO setpoint of either 30% of 50% (Lee et al. 2003). One of the main findings of that study was the specific growth rate being higher for the narrow DO band (10 − 30% DO) than for the wider DO band (10 − 50% DO) due to the more frequent feeding, thereby corroborating the findings of the current study. These findings are further corroborated by a study by Jia et al., where an online model-based DO-stat control was implemented in the methanol induction stage, to maintain narrower and more stable DO bandwidths (± 20%) (Jia et al. 2022). The implementation of this control strategy increased the methanol feed, which notably increased cell concentrations as well as heterologous protein production levels (Jia et al. 2022).

No residual glycerol was detected during the fed-batch cultivations, which implied that the cultures were maintained under carbon-limited conditions and that biomass growth was not inhibited by substrate accumulation. However, the constant feed fermentations required supplementation with pure O_2_ throughout the fed-batch phase to maintain the DO at the required level of 30% of saturation in order to ensure that a dual limitation (carbon and oxygen) was not imposed (Fig. 6b). On the other hand, the DO-stat fermentation did not require pure O_2_ supplementation, except for a brief spike near the end of the batch phase where the culture exhibited rapid growth (Fig. 6a). Due to the poor DO-stat control, the culture growth rate was reduced (maximum biomass concentration of 119.73 ± 1.99 g/L after 155 h for DO-stat and 133.86 ± 3.92 g/L after 59 h for constant feed) such that the rate of oxygen consumption remained low and O_2_ supplementation was not required.

In this study, the final biomass concentration, biomass productivity as well as the growth rate were all higher in the constant feed fermentations compared to DO-stat fermentations; however, this did not translate into higher volumetric activity (Table 2). According to literature, protein production in *P. pastoris* can either be growth associated (Cunha et al. 2004), partially growth associated (Kobayashi et al. 2000) or growth dissociated (Potgieter et al. 2010). However, proteins expressed under control of the *GAP* promoter are generally accepted to be primarily growth-associated, i.e., the rate of product formation and product titre are correlated to the culture growth rate (Rebnegger et al. 2014; Looser et al. 2015) and biomass concentration (Pal et al. 2006). An increase in the specific growth rate leads to an increase in the glycolytic flux, higher transcription levels of the gene under *GAP* promoter control (Garcia-Ortega et al. 2016) as well as increases in the specific rate of protein secretion (Rebnegger et al. 2014). This is contradictory to the finding in this study and could possibly be attributed to saturation of the secretory pathways and subsequent degradation of the accumulated intracellular product (Idiris et al. 2010; Kuo et al. 2015), in spite of increased levels of growth-associated protein expression at higher growth rates. However, a study by Li et al. showed that the production of herring antifreeze protein increased with decreasing growth rate as a result of a decrease in fermentation temperature, which was postulated to be due to improved protein folding (Li et al. 2001). This could also explain the higher volumetric activity for the GAPfopA and GAPfopA_V1 strains at the lower growth rates in this study. Collectively, this highlights that it is not always possible to predict protein production solely based on biomass growth, prompting case by case predictions instead.

The DO-stat fermentation produced a higher maximum volumetric activity than the constant feed fermentation, but the shorter process time of the constant feed fermentation resulted in a higher volumetric productivity (maximum of 23.96 ± 0.44 × 10^3^ U/L/h for constant feed and 13.74 ± 5.27 × 10^3^ U/L/h for DO-stat). Menéndez et al. recombinantly expressed and produced the β-fructosidase enzyme from *Thermotoga maritima* in *P. pastoris* using a constant feed fed-batch fermentation and achieved an enzyme productivity of 3347 U/L/h (Menéndez et al. 2013). Martínez et al. produced the same enzyme in *P. pastoris* using constant feed as well as exponential feed fed-batch fermentation, and achieved volumetric productivities of 5735 U/L/h and 4 662 U/L/h, respectively (Martínez et al. 2014). A higher volumetric productivity is advantageous for manufacturing processes as it results in a higher enzyme production per unit volume of bioreactor – this in turn translates into more efficient and cost-effective application of equipment capital (Looser et al. 2015).

## Conclusions

The GAPfopA and GAPfopA_V1 enzymes were successfully expressed and produced by *P. pastoris* to high titre using fed-batch culture. The DO-stat fermentation was characterised by poor DO control and slow substrate feed rates, which had a marked effect on culture growth in terms of biomass accumulation. Nonetheless, despite this inferior performance, the DO-stat cultures outperformed the constant feed cultures as evident from higher volumetric enzyme activity. However, the extended process time to reach this higher level of enzyme production, as evident from SDS-PAGE analysis, resulted in lower volumetric enzyme productivity in the DO-stat cultures. Finally, the constant feed method exhibited better reproducibility as well as higher volumetric enzyme productivity despite a marginally lower enzyme production.

## Data Availability

No datasets were generated or analysed during the current study.
